# Evolving media use in early childhood: parental motivations and their impact on child development

**DOI:** 10.3389/fpsyg.2026.1721813

**Published:** 2026-02-04

**Authors:** Sarah M. Coyne, Lara A. Maximiano Almeida, Jane Shawcroft, Cambria Siddoway, Talise Hirschi

**Affiliations:** 1 School of Family Life, Brigham Young University, Provo, UT, United States; 2 Department of Communications, The Ohio State University, Columbus, OH, United States

**Keywords:** calm, early childhood, media, motivations, parenting, reasons

## Abstract

Media use during early childhood is common and is related to a range of both positive and negative outcomes for children. However, the motivations for why parents allow children to use media have rarely been examined, particularly over time. The current study examines the growth of four different media motivations across four years in early childhood: using media to calm children down, to educate, for enjoyment, and to keep children busy. Additionally, predictors and outcomes of the growth of media motivations were examined. Participants included 467 infants (*M* age = 17.77 months at the initial time point) and their caregivers. Parents completed questionnaires once a year for 4 years on their motivations for giving their children media and on child internalizing and externalizing behaviors. Using media to keep children calm was moderate at the initial time point and decreased over time. Conversely, the other three types of media motivations increased steadily over time, particularly using media because children enjoyed it. Initial levels of motivations to keep children calm and to keep children busy were both related to externalizing behaviors 4 years later. These results suggest that media motivations change over the course of early childhood and are differentially related to child behavioral outcomes over time.

## Introduction

Media and technology are normative parts of early childhood. Part of the ubiquity of media during childhood is due to the various purposes media can have for parents of young children. These include supporting children’s education (Chong et al., 2023), for children’s entertainment or enjoyment (Hinkley and McCann, 2018), to occupy children so that parents can accomplish other tasks (Nikken, 2019), or to help regulate children’s emotions (Shawcroft et al., 2025; Radesky et al., 2016). As such, parents have a variety of motivations for encouraging children’s media use (Geurts et al., 2022; Nikken, 2019). Despite the variety of motivations parents may have for facilitating their children’s media use, much of the research emphasis has been on the frequency or content of media and technology use by young children, and little is known about the ways parents’ motivations for their children’s media use evolves over time, or developmental implications (i.e., socioemotional outcomes) of these different motivations for young children.

The importance of understanding parents’ motivations for facilitating their children’s media use is highlighted by the DREAMER model (Barr et al., 2024), which conceptualizes media effects as emerging from the dynamic interplay of individual characteristics, relational processes, and broader ecological contexts. The DREAMER model emphasizes the importance of proximal media use contexts. It focuses on the when and why (including parental media motivations), rather than just the amount of time media is being used, as important factors for child and family outcomes. The model integrates concepts from family systems theory, relational theory, ecological systems theory and transactional and dialectical models (Barr et al., 2024) that help understand how parental motivations for children’s media use are a central mechanism through which media becomes embedded in family routines, with the potential to shape child development.

Most research on parental media motivations are either cross-sectional or qualitative (see Geurts et al., 2022). However, parental motivations for giving children media likely change over time. Thus, the current study examines longitudinal trajectories of four types of media motivations (to educate, because the child enjoys media, to keep the child busy, and to calm the child) across a 4-year period during early childhood (spanning when children are ages 1.5–4.5 years old; RQ1) using the DREAMER model. Additionally, we examine both predictors and outcomes of media motivations over time (RQ2). Specifically, we examine how child externalizing and internalizing behaviors, parent education and age, and children’s TV viewing time all predict the intercept and slope of media motivations, as well as the association between the intercept and slope of media motivations on children’s later internalizing and externalizing behaviors (when children are 6.5 years old). We hypothesize that use of media to calm children would decrease as children age and their capacity to self-regulate grows. We also hypothesize that using media to keep children calm would be related to increased internalizing and externalizing behavior at Wave 6, when children are 6.5 years old. Little research has examined other media motivations and the links to children’s behaviors, so these associations remain exploratory.

## Methods

Data from this paper came from Project MEDIA, a longitudinal study focused on long-term effects of media on child development. Starting in 2017 (Wave 1) 500 primary caregivers and their infants under the age of one were recruited for participation in this study. Participants were recruited through flyers sent in the mail to the participants’ homes through the Colorado Office of Health and Vital Records, which identified all in the local area who had given birth in the past year (27.5% of the 500 families). Research assistants visited potential participants’ homes to invite participation and were able to collect in-home data for this project from 66% of the participants who had a child in the home under the age of one at Wave 1. Participants were also recruited using flyers in pediatrician offices, free clinics, social services offices, businesses focused on entertainment for young children, public parks and play spaces, and a referral from a friend who participated (22.7%). Finally, 49.8% of the sample was recruited through an external data collection company.

The current paper uses data from Waves 2 through 7 (referred to in this manuscript as Wave 1 through 6 for reader clarity), when media motivations were introduced in the study. Data for this study consists of 467 child/parent dyads. Children (51% male) were approximately 17.77 months at the initial time point (SD = 3.66 months) and were primarily White (68%), Hispanic/Latine (8%), Black (8%), multi-racial (16%) or other (3%). Families needed at least one wave of data to participate and there was an 89% retention rate across all four waves used in the growth curves (Waves 1 through 4). Participants completed a series of questionnaires on motivations for media use and child behavior and there was approximately a 1-year time lag between each wave of data collection.

### Measures

#### Motivations for media use; Waves 1–4

Parents’ motivations for children’s media use were measured using four items created for Project MEDIA, asking parents to rate how often they allow their children to use media for the following reasons: to educate, keep their child busy, because their child enjoys media, and to calm their child down. Although enjoyment is more child-driven than the other motivations, previous research shows that parents often allow media use because their child finds it entertaining (Chong et al., 2023; Hinkley and McCann, 2018; Livingstone et al., 2015). For this reason, we included the item “because my child enjoys using the device” as a parent-relevant motivation when thinking of media decisions. Responses were given on a seven-point Likert scale ranging from 1 (never) to 7 (several times per day). Items were: “to educate my child,” “to keep my child busy while I get things done,” “because my child enjoys using the device,” and “to calm my child down when they are upset.” Higher scores indicate that media use was motivated by that purpose more often.

#### Children internalizing and externalizing behavior: Wave 1 and 6

Child’s internalizing and externalizing behavior was assessed using parental report of the Child Behavior Checklist (CBCL; Achenbach and Ruffle, 2000). Parents responded to the 100-item measure on a three-point Likert scale, with response options ranging from 0 (not true, as far as you know) to 2 (very true or often true). These items were summed to create two overall measures of children’s internalizing (Wave 1: α = 0.75; Wave 6: α = 0.87) and externalizing (Wave 1: α = 0.89; Wave 6: α = 0.92) behaviors. Example items for the internalizing behaviors subscale include “feelings hurt easily,” “acts too young for age,” and “too upset by separation from parents” and for externalizing; “hits other,” “can’t sit still,” and “punishment doesn’t change his/her misbehavior.” Higher scores indicate greater behavior problems in each category.

#### Additional covariates

In addition, we assessed children’s TV time, parent education, and parent age. Parent education was measured on a 1–8 point scale with 1 = no formal school, 2 = Elementary/primary school, 3 = Middle school, 4 = High school or Equivalent, 5 = Some college or vocational degree, 6 = Bachelor’s degree, 7 = Master’s degree, 8 = Advanced professional degree (e.g., MD, JD, PhD). Television time was measured using a single item asking parents to report on the amount of time their child spent on a typical day viewing television or film content on any device (e.g., television set, tablet, phone, computer). This was measured on a 1 (never) to 8 (more than five hours a day) Likert scale.

### Plan of analysis

To answer RQ1, we specified linear growth curves of each of the media motivations (education, keeping child busy, because the child enjoys media, and to calm the child down) across the four waves in Mplus (v. 8.8). These grown curves were specified as separate models. Quadratic terms were added and retained in the models if significant. To examine RQ2, H1 and H2, parent age and education as well as child internalizing and externalizing behaviors and television viewing at Wave 1 were added as predictors for the intercept and slopes of media motivations. The intercepts and slopes of media motivations were specified as predictors of child internalizing and externalizing behaviors at Wave 4.

## Results

**FIGURE 1:**
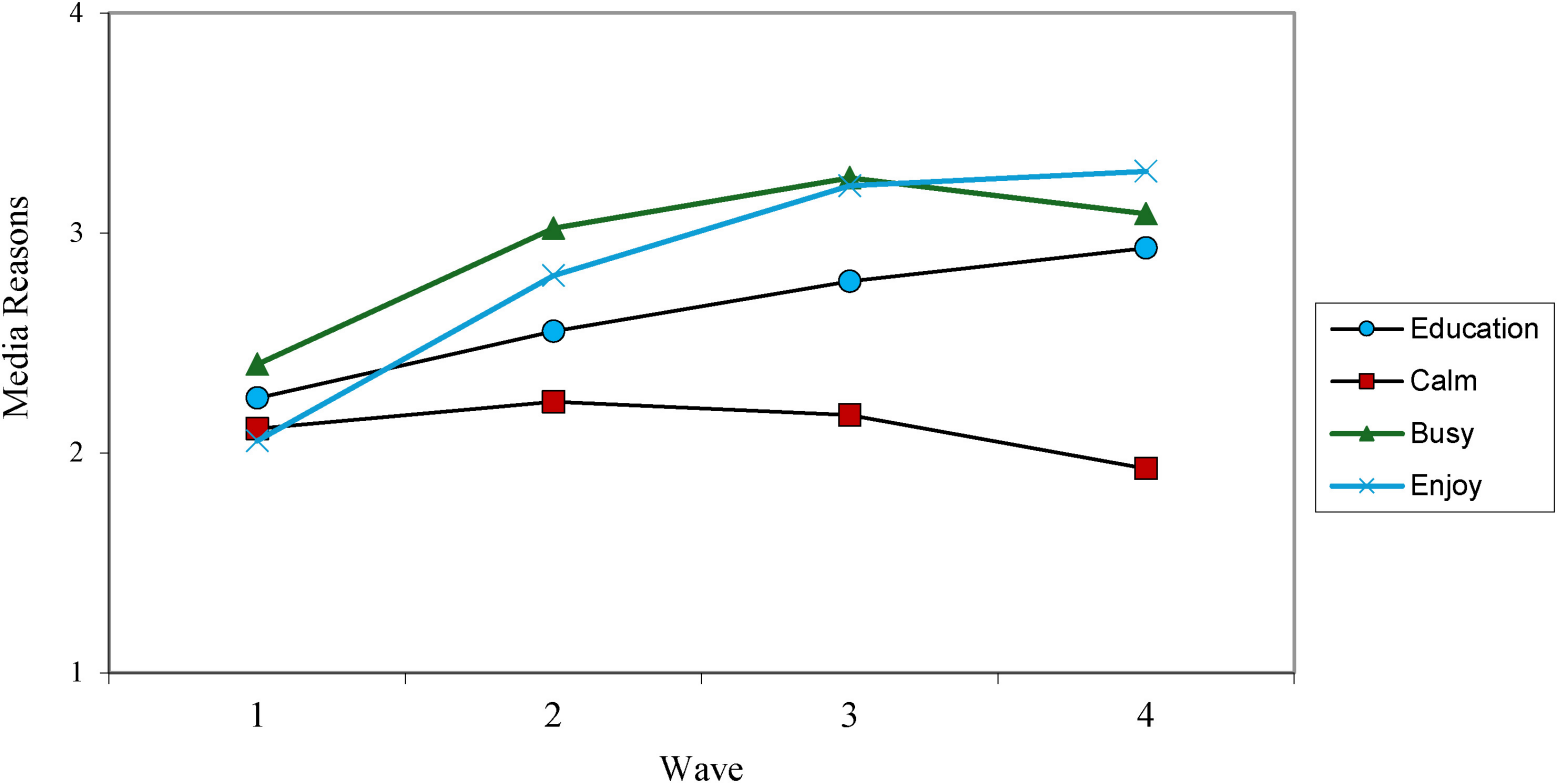
Growth trajectories for each media motivation.

Model fit of the four growth curves of media motivations with predictors and outcomes was acceptable (see Table 1), with the CFI ranging across the models between 0.88 and 0.91, the RMSEA ranging between 0.07 and 0.09, and the SRMR ranging between 0.06 and 0.11. For education motivations, there was an intercept of 2.18, S.E. = 0.06, *p* < 0.001. This can be interpreted as parents in our sample on average when children were 1.5 years old, reporting educational purposes motivated children’s media use between “A little bit” (response level 2) to “quite a bit” (response level 3). We also found a positive linear slope, *b* = 0.65, S.E. = 0.07, *p* < 0.001, indicating parents’ educational motivations increased as children aged. In addition, we found evidence of a significant quadratic term, *b* = −0.15, S.E. = 0.02, *p* < 0.001 (see Figure 1).

**TABLE 1:** Model fit for all growth models.

Media motivation	Model fit
Education	χ^2^ (28) = 59.71, *p <* 0.001, CFI = 0.91, TLI = 0.85, RMSEA = 0.07, SRMR = 0.06
Calm	χ^2^ (28) = 74.42, *p <* 0.001, CFI = 0.89, TLI = 0.82, RMSEA = 0.09, SRMR = 0.10
Busy	χ^2^ (28) = 69.46, *p <* 0.001, CFI = 0.89, TLI = 0.82, RMSEA = 0.08, SRMR = 0.10
Enjoy	χ^2^ (28) = 71.36, *p <* 0.001, CFI = 0.88, TLI = 0.80, RMSEA = 0.09, SRMR = 0.11

Using media to keep children calm had an intercept of 2.11, S.E. = 0.06, *p* < 0.001. This is somewhat lower than educational motivations and indicates that on average parents at Wave 1 used media to calm children between “a little bit” (response level 2) and “quite a bit” (response level 3). We found a significant positive linear slope, *b* = 0.21, S.E. = 0.07, *p* = 0.001, and quadratic term, *b* = −0.09, S.E. = 0.02, *p* < 0.001.

Using media to keep children busy had the highest intercept at Wave 1 compared to other motivations, with a mean of 2.40, S.E. = 0.06, *p <* 0.001 (still however, falling between “a little bit” and “quite a bit”). This motivation also had the steepest linear slope of the motivations, *b* = 0.82, S.E. = 0.07, *p* < 0.001, and a significant quadratic term, *b* = −0.20, S.E. = 0.02, *p* < 0.001.

Finally, media use motivated by children’s enjoyment of media had the lowest intercept of all the media motivations at 2.10, S.E. = 0.06, *p* < 0.001. There was also a positive linear slope, *b* = 0.92, S.E. = 0.08, *p* < 0.001, and significant quadratic term, *b* = −0.17, S.E. = 0.02, *p* < 0.001.

Pertaining to RQ2, child externalizing behaviors at Wave 4 was positively related to the intercept of using media to keep children busy, β = 0.23, S.E. = 0.09, *p* = 0.010, as well as the intercept of using media to calm children, β = 0.19, S.E. = 0.10, *p* = 0.045. There were no other significant associations between the intercept and slope of media motivations and child internalizing and externalizing behaviors at Wave 4.

Children’s TV time was positively related to the intercept of all media motivations and negatively related to the slope of using media to calm children (see Table 2). This means that across all four motivations for children’s media use, higher levels of TV time were related to more of that media motivations at Wave 1, but less steep increases in using media to calm children. In addition, children’s early externalizing behaviors were positively related to the intercept of using media to keep children busy (although this relationship was only marginally significant), β = 0.24, S.E. = 0.12, *p* = 0.051, and parent education was negatively related to the intercept of using media to calm children, β = −0.22 S.E. = 0.10, *p* = 0.022, as well as educational purposes, β = 0.01, S.E. = 0.10, *p* = 0.029.

**TABLE 2:** Predictors and outcomes of the growth of media motivations.

Motivation	Child predictors W1									Parent predictors W1						Child outcomes W4					
	Externalizing behaviors			Internalizing behaviors			TV time			Age			Education			Externalizing behaviors			Internalizing behaviors		
	β	S.E.	*P*	β	S.E.	*P*	β	S.E.	*P*	β	S.E.	*P*	β	S.E.	*P*	β	S.E.	*P*	β	S.E.	*P*
**Education**																					
Intercept	0.16	0.13	0.218	0.02	0.08	0.777	0.52	0.08	<0.001	0.01	0.09	0.939	−0.21	0.10	0.029	−0.04	0.28	0.891	0.02	0.25	0.925
Slope	−0.91	1.11	0.410	−0.40	0.46	0.387	−0.21	0.91	0.819	−0.04	0.28	0.890	0.09	0.27	0.748	−0.38	0.47	0.412	−0.33	0.40	0.410
**Calm**																					
Intercept	0.12	0.11	0.279	0.09	0.10	0.351	0.56	0.08	<0.001	−0.02	0.10	0.832	−0.22	0.10	0.022	0.19	0.10	0.045	0.15	0.11	0.166
Slope	−0.02	0.11	0.842	0.06	0.10	0.577	−0.26	0.09	0.003	0.02	0.10	0.884	0.01	0.09	0.889	−0.06	0.08	0.430	−0.10	0.07	0.162
**Busy**																					
Intercept	0.24	0.12	0.051	0.01	0.11	0.928	0.63	0.10	<0.001	−0.04	0.09	0.684	−0.02	0.10	0.857	0.23	0.09	0.010	0.15	0.11	0.144
Slope	−0.05	0.24	0.829	<−0.01	0.17	0.997	−0.32	0.18	0.071	0.04	0.13	0.786	0.11	0.12	0.340	−0.07	0.22	0.758	−0.08	0.21	0.685
**Enjoy**																					
Intercept	0.03	0.16	0.856	<0.01	0.11	>0.999	0.57	0.09	<0.001	−0.11	0.11	0.323	−0.01	0.11	0.903	0.04	0.13	0.759	0.02	0.132	0.868
Slope	0.13	0.25	0.592	−0.03	0.13	0.851	−0.31	0.19	0.105	0.17	0.17	0.341	−0.12	0.15	0.423	0.10	0.20	0.620	0.07	0.16	0.675

## Discussion

This study found that out of four parental motivations for child media use (educate child, keep child busy, child enjoyment, calm child down) during early childhood (1.5–4.5 years), media to keep children busy had the highest initial use and the steepest increase over time. Meanwhile, media for calming had the slightest increase before starting to decrease; media for education increased steadily over the years before stabilizing, and media for enjoyment was the only one that increased continuously during the last year while others decreased.

Following the DREAMER perspective (Barr et al., 2024), these results show how different parental motivations, more than just screen time, may be associated with a significant role in child developmental outcomes over time. This was further supported considering how initial media motivations were predicted by early externalizing behaviors and sociodemographic factors such as parent education and initial TV use. However, as mentioned in the DREAMER framework, bidirectional effects might also play a role in these relationships since media parental motivations might reflect both influences from and responses to of child characteristics. Child behavior has the ability to affect parenting behaviors regarding media, which could then shape child development.

Notably, mean levels of media use for calming purposes initially increased until around 2.5 years old but decreased afterwards. One possible explanation is that children are still developing self-regulation skills at this age and rely heavily on their parents for co-regulation. This reliance may be associated with parents turning more often to immediate solutions like media to soothe their children, particularly in social settings such as restaurants and grocery stores (e.g., de Paz-Cantos et al., 2024; Geurts et al., 2022). The decline after age 2.5 is consistent with our hypothesis and may reflect children’s development of more independent coping skills, as well as parents’ growing confidence in managing their child’s emotional states (Russell et al., 2021). Children may also become accustomed to using media as a soothing tool and begin to enjoy it, which could prompt parents to shift their ratings from “calming down” to “enjoyment.” This pattern underscores the dynamic nature of media use across development and aligns with the DREAMER model (Barr et al., 2024), which highlights the importance of timing and context in shaping media’s role in family life.

“Enjoyment” showed the most dramatic growth, suggesting a developmental shift as children become more engaged and begin to express their preferences. “Education” also emerged as a common motivation for parents, particularly in early childhood (Nevski and Siibak, 2020). While our study found that this motivation increased steadily before leveling off, prior research indicates that parents often label content as educational even when it is not, highlighting discrepancies between parental and researcher classifications (Baek et al., 2013).

We also found that initial levels of using media to keep children calm were associated with higher levels of externalizing behavior at later waves, but not at the initial time point. One possibility is that when media is used to help children manage their emotions over long periods of time, they may have fewer chances to practice regulating those emotions on their own. Conversely, using media to keep children busy was related to externalizing behaviors at both the initial (although only marginally significant) and final waves of data. Children in these situations may simply have fewer opportunities to practice staying focused or entertaining themselves, which may be associated with difficulties to manage without media. This pattern also suggests that media may serve a practical, day-to-day function in many families’ routines (Geurts et al., 2022). Future studies could examine how regular use of media during predictable activities or times of day versus irregular use impacts children’s socioemotional behaviors.

It is important to acknowledge some of the limitations of this study including that it was parental report, our sample was primarily white, and socio-economic factors were not controlled for. Similarly, potential confounding variables such as parental stress, mental health, parent/child relationship, parenting style or household chaos were not controlled for. Both the media motivation scale and the Child Behavior Checklist (CBCL) were filled out by parents, which could introduce shared method variance or possible reporting bias and could create a stronger relationship between our predictor and outcome variables than truly exists. Although our longitudinal design provides valuable information, bidirectional parent–child processes emphasized in the DREAMER framework were not accounted for. Therefore, child behaviors may both influence and be influenced by parental media motivations. Additionally, there are likely other parental motivations for children’s media use that we did not capture in the current study. The addition of these variables may increase consistency for results around child behavior and may better explain associations over time. Furthermore, the scale that was used to measure media motivations only consisted of a single item for each media motivation. Future studies could create multi-itemed scales to measure media motivations and reduce potential concerns about measurement reliability and construct validity. Future studies should also consider how cultural values or norms may impact a parent’s motivation in giving their child media, and they should also study different types of media content, the context of use (e.g., time of the day, co-viewing or not), and how the content and context are influenced or interact with parents’ motivations.

This study highlights the importance of considering why parents turn to media and promoting alternative strategies for calming and occupying children that may be more beneficial. Pediatricians, educators, and digital literacy professionals should also recognize that parental motivations shift over time, underscoring the need to tailor guidance to the child’s developmental stage. Overall, these findings provide a more holistic understanding of early childhood media use, showing that parental motivations are dynamic and shaped by both parent and child characteristics, and pointing to the need for nuanced research and interventions that reflect the evolving role of media in family life.

## Data Availability

The raw data supporting the conclusions of this article will be made available by the authors, without undue reservation.
